# Hospitals and Street Noises

**Published:** 1911-10-21

**Authors:** J. J. Barron

**Affiliations:** Secretary-Superintendent Royal Infirmary, Bradford.


*To the Editor of* The Hospital.

### HOSPITALS AND STREET NOISES.

*To the Editor of* The Hospital.

Sir,—I was much interested in your remarks on the attempt being made in Sheffield to reduce the noise from traffic in the streets adjacent to the Royal Hospital, and I would like to draw your attention to the fact that all Northern cities are not so much behind London and the Southern county towns in this respect as you seem to indicate. In Bradford the Royal Infirmary has a main road with tram-lines on the west side, and on the east there is another fairly busy roadway. In order to relieve the patients in the wards from street noises the City Council many years ago laid wood paving on both the roads mentioned alongside the infirmary grounds and buildings, and this has done a good deal towards reducing the noise of street traffic.—I am, yours truly,

J. J. Barron, Secretary-Superintendent

October 14. Royal Infirmary, Bradford.

